# Collecting and using patient experience data: Caution, commitment and consistency are needed

**DOI:** 10.1111/hex.12863

**Published:** 2019-01-29

**Authors:** Mary Chambers, Joseph LeMaster

**Affiliations:** ^1^ Faculty of Health, Social Care and Education Kingston and St George’s University of London Cranmer Terrace London SW17 ORE UK; ^2^ Research Division Department of Family Medicine School of Medicine University of Kansas Kansas City KS 66160 USA

Data on patient and public involvement vary in the extent and consistency in which they are collected and used across contexts (clinical, research and service improvement). Collection and analysis of patient experience data in research and in service development/improvement have different purposes.

Such data may not always be translated or utilized well when the aim is service improvement.^1^, ^2^, ^3^ The reasons for this include the following: how the data are collected, and resources or time provided by healthߚcare systems to use the data in serviceߚrelated improvement work or research.^4^, ^5^ Tokenistic participatory approaches limit the appropriate use of patientߚreported “lived” experience (emic) data.^6^

This issue of HEX illustrates various ways in which data on patient experiences and public involvement are collected and used. These studies highlight the importance of respecting and valuing patients’ “voices,” as well as barriers and facilitators to doing so.

The two review papers Pii et al and Petkovic et al, whilst using different methodological approaches, both indicate the need to attune studies to the needs and participation of patients and others from marginalized or socioeconomically disadvantaged groups. Pii et al describe a systematic review addressing questions on the stages, methods and challenges of involving patients with a cancer diagnosis in the research process. Concerns about the collection of sociodemographic data in healthߚcare settings were outlined by Petkovic et al. Whilst patients acknowledged the importance of providing data about their experiences, there were concerns about how these were collected, and data requested regarding income, religion and ethnicity were of particular concern.

Patients accept that providing data about their experiences is important (being mindful of the caveats above), and in developing areas such as selfߚcare/management, this is particularly so. As individuals across the demographic trajectory take greater responsibility for their own care, their voices need to be heard and different approaches may be needed to capture these, as reflected in the papers of Bossy et al and Sheridan et al. Notwithstanding this, as highlighted by Sheridan et al, a strong relationship and good continuity with healthߚcare professionals is key to supporting patient outcomes, such as selfߚmanagement.

As the digital environment takes a more prominent role across all sectors of health care, the papers by Burrows et al, Ross et al and Dennehy et al indicate the nature and variety of such engagement and also highlight how patient and public can contribute to the way experience data are collected.

Coߚproduction is a developing area aligned to patient and public involvement with standards that have only recently been defined, and with respect to research not explicitly service improvement.^7^ The papers authored by HollandߚHart et al and Louch et al explore the barriers and facilitators associated with coߚproduction as well as difficulties associated with implementation. Even though patients, public and professionals work together to develop initiatives to ensure the valuing and respecting of data collected from both patients and public, these authors suggest that some professionals have concerns about how such data will be used in organizational decision making. Related to the use of patient and public generated data, Sheard et al point out the ethical responsibility to train staff to use patient experience data meaningfully and provide them with data in a way that highlights actionable items, that is, “quality over quantity.”

In summary, the studies in this issue considered a variety of approaches to the collection and use of data pertaining to patient and public involvement and patient experiences of health care.
